# Oleuropein and Cancer Chemoprevention: The Link is Hot

**DOI:** 10.3390/molecules22050705

**Published:** 2017-04-29

**Authors:** Ammad Ahmad Farooqi, Sundas Fayyaz, Ana Sanches Silva, Antoni Sureda, Seyed Fazel Nabavi, Andrei Mocan, Seyed Mohammad Nabavi, Anupam Bishayee

**Affiliations:** 1 Laboratory for Translational Oncology and Personalized Medicine, Rashid Latif Medical College, Lahore 54000, Pakistan; ammadfarooqi@rlmclahore.com (A.A.F.); sundas.khan23@yahoo.com (S.F.); 2 Department of Food and Nutrition, National Institute of Health Dr. Ricardo Jorge, I.P., Av. Padre Cruz, 1649-016 Lisbon, Portugal; anateress@gmail.com; 3 Center for Study in Animal Science, Praça Gomes Teixeira, Apartado 55142, 4051-401 Porto, Portugal; 4 Research Group on Community Nutrition and Oxidative Stress and CIBEROBN—Physiopathology of Obesity and Nutrition, University of Balearic Islands, Palma de Mallorca E-07122, Balearic Islands, Spain; tosugo@hotmail.com; 5 Applied Biotechnology Research Center, Baqiyatallah University of Medical Sciences, Tehran 1435916471, Iran; Nabavisf@gmail.com; 6 Department of Pharmaceutical Botany, Iuliu Hațieganu University of Medicine and Pharmacy, 400012 Cluj-Napoca, Romania; mocan.andrei@umfcluj.ro; 7 ICHAT and Institute for Life Sciences, University of Agricultural Sciences and Veterinary Medicine, Calea Mănăştur 3-5, 400372 Cluj-Napoca, Romania; 8 Department of Pharmaceutical Sciences, College of Pharmacy, Larkin University, Miami, FL 33169, USA

**Keywords:** cancer, chemopreventive effect, oleuropein, olive oil

## Abstract

Cancer comprises a collection of related diseases characterized by the existence of altered cellular pathways resulting in an abnormal tendency for uncontrolled growth. A broad spectrum, coordinated, and personalized approach focused on targeting diverse oncogenic pathways with low toxicity and economic natural compounds can provide a real benefit as a chemopreventive and/or treatment of this complex disease. Oleuropein, a bioactive phenolic compound mainly present in olive oil and other natural sources, has been reported to modulate several oncogenic signalling pathways. This review presents and critically discusses the available literature about the anticancer and onco-suppressive activity of oleuropein and the underlying molecular mechanisms implicated in the anticarcinogenic and therapeutic effects. The existence of limitations and the promising perspectives of research on this phenolic compound are also critically analyzed and discussed.

## 1. Introduction

Natural products obtained from various sources have been a source of inspiration for medicinal chemists and correspond to a significant reserve for the identification of new drugs. Among different sources of natural products, medicinal and edible plants have been precious sources of therapeutic agents for millennia, and numerous medicines generally used today are based on natural products from plants or their derivatives [1,2,3]. Medicinal plants and vegetables together with food products derived from them are important sources of natural compounds that have proven their beneficial effects for health by preventing or curing diseases [4,5]. Referring to medicinal plants, the bioactive natural compounds can ultimately be developed as pharmaceuticals [4,6,7,8].

Natural products from various sources have been used for the prevention or treatment of several chronic ailments for centuries. In this frame, cancer is a growing health concern worldwide, especially associated with the progressive increase in life expectancy, increased urbanization, the shift towards a more sedentary lifestyle and subsequent transformation of the environmental conditions [9,10]. Moreover, an abundance of mechanistic information has recently been presented about how phytochemicals derived from dietary sources have reputed chemopreventive properties that interfere with the initiation, promotion, and progression of tumor. Chemopreventive phytochemicals often have similar or identical mechanisms with molecularly targeted chemotherapeutic agents, and may therefore serve as alternative or complementary coadjuvant to conventional antitumoral therapies. In a similar manner to synthetic chemotherapeutical agents, chemopreventive phytochemicals confound events from different signaling pathways involved in tumor growth or invasion and may ultimately act as antineoplastic agents [11,12].

Additionally, a large amount of epidemiological evidence suggests that a habitual intake of vegetables, fruits, and herbal products is linked with a reduction in the risk of suffering from chronic diseases such as cardio-metabolic syndrome and cancer. This assumption is at least in part sustained by the presence of several important phytochemicals in plant-based foods [13,14]. Olive oil, especially extra virgin olive oil, derived from the olive tree (*Olea europaea*) is highly appreciated for its taste and flavor, but also for its health benefits. The intriguing properties of this functional food are ascribed to its adequate fatty acid profile and phenolic composition [15]. Among bioactive components of olive oil, phenolic compounds have been extensively investigated and their occurrence and health claims are well documented by many studies [15]. This review presents comprehensive information about the chemistry, dietary sources, bioavailability, and anticancer effects of oleuropein, one of the most abundant bioactive components contained in the olive fruit of the Oleaceae family, with a special emphasis on molecular mechanisms and involved signaling pathways.

## 2. Chemistry

**Figure 1:**
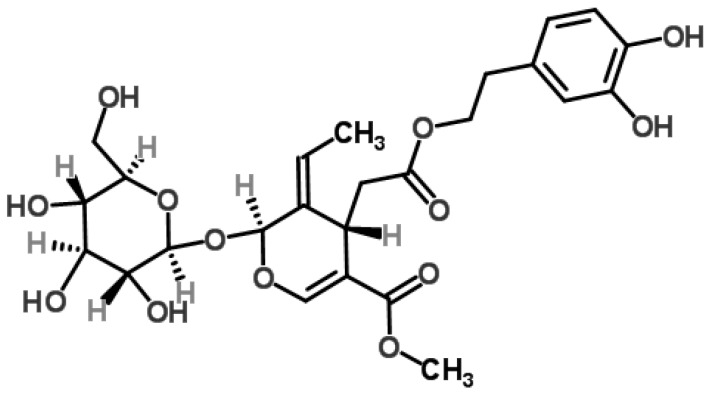
Chemical structure of oleuropein [19].

Oleuropein is a member of the secoiridoids that belongs to the class of coumarin-like components. It is a secoiridoid glycoside that is abundant in plants of the family Oleaceas, Gentianales, Cornales [15]. It is one of the most common active compounds in the leaves of olive tree (*Olea europaea* L.), the use of which dates back to 6000 BCE according to archaeological evidence [16]. In fact, olive leaves were used by ancient Egyptians to mummify pharaohs in the classical era and later, and in folk medicine to treat fevers, colic, alopecia, sciatica, paralysis, rheumatic pain, and hypertension [17,18]. Olives are used to produce extra-virgin olive oil, the main lipid source in the “Mediterranean diet” associated with several health benefits, including low incidence of cardiovascular diseases. These benefits are generally related to the favorable fatty acid (FA) based composition of extra virgin olive oil and the occurrence of diverse minor constituents (e.g., carotenoids and polyphenols) responsible for the unique taste and flavor [17]. Extra-virgin olive oil is obtained by crushing olives using mechanical or other physical procedures that do not imply any alteration in the oil composition. Oleuropein consists of hydroxytyrosol (3,4′-dihydroxyphenylethanol), elenolic acid, and glucose (Figure 1). Table 1 presents various chemical and physical properties of oleuropein. In the tree, oleuropein is believed to confer resistance to insect and infections [17].

## 3. Sources

Oleuropein, a characteristic biophenol present in the plants of Oleaceae family, is responsible for the bitterness of olive fruits [20]. Phenols of olives are also responsible for the resistance of olive oil to oxidative rancidity [17]. Olive biophenols greatly change depending on variety, species, ripening stages and edaphoclimatic conditions during development [21,22]. For instance, demethyloleuropein was reported as a potential varietal marker because it was not found in all the varieties [17,23]. Several methods can be used to determine oleuropein in different parts of the *Olea europaea* (fruits, leaves, stems, and roots) or its products (pomace, olive oil, and alperujo) [24], including infrared spectroscopy [25], voltammetry [26], capillary electrophoresis with UV detection [27], capillary electrophoresis-electrospray ionization mass spectrometry (CZE-ESI-MS) [28], gas chromatography-mass spectrometry (GC-CI-MS) [29,30], or high performance liquid chromatography (HPLC), which can be equipped with several detectors such as UV detector (HPLC-UV) [20,31,32], diode array detector (HPLC-DAD) [24,33], electrospray ionization tandem mass spectrometry (HPLC-ESI-MS/MS) [21,33,34,35], ion trap multiple mass spectrometry (IT-MSn), and electrospray time-of-flight mass spectrometry (HPLC-ESI-TOF-MS) [36]. Table 2 presents the concentration of oleuropein in different parts of the olive plant or in different products (e.g., olive pomace and extra virgin olive oil).

Although oleuropein is found in all parts of the olive tree and olive fruits (peel, pulp and seed), the highest amount is found in olive leaves. Small fruit cultivars are associated with high oleuropein content and large fruit cultivars with low oleuropein content [17]. The analysis of oleuropein in olive leaves revealed that green leaves presented higher levels of oleuropein than green-yellowish leaves and these presented higher content than yellow leaves [37]. A study developed by Ortega-García et al. [31,38] using the Picual variety reported that oleuropein decreases in fruits while increases in leaves over olive ripening process.

## 4. Biosynthesis and Bioavailability

In *Olea europaea*, biosynthesis of Oleuropein is complex and still not very well elucidated. Moreover, it may vary according to the species and the season of the year [21]. It is believed that oleuropein is biosynthesized from mevalonic acid through intricate metabolic pathways. It involves the formation of carbocyclic iridoid precursors (deoxyloganic acid, 7-ketologanin, 7-epi-loganic acid, and 7-ketologanic acid) although their order may vary according to the species and season of the year.

According to Damtoft et al. [47,48], 7-ketologanin is a previous intermediate of the oleoside-11-methyl ester and its transformation is more likely to occur in a single-step process by a Baeyer-Villiger type intermediate, although other processes may be possible. The final stages of the synthesis of oleuropein could be due to the direct transformation of 7-ketologanin into oleoside-11-methyl ester and the subsequent conversion to 7-β-1-d-glucopyranosyl-11-methyl oleoside that is esterified with tyrosol to give ligstroside. A hydroxylation reaction occurs, leading to oleuropein formation.

During the process of maturation, crushing, malaxing, and manipulation of olives, oleuropein may undergo biotransformation in its respective aglycon by endogeneous β-glucosidades [29]. The hydrolysis of oleuropein aglycone can give rise to many forms of elenolic acid and hydroxytyrosol characterised for their radical scavenging capability [42]. During the milling and kneading, endogenous oxidoreductases (e.g., polyphenoloxidase, PPO, peroxidase, and POX) are also suggested to have a distinct role in the olive oil extraction procedure via favouring phenolic oxidation [49].

According to a study by Vissers et al. [50,51] on bioavailability of olive oil phenols in humans, their absorption rate is more than 55–66 mol % and elimination rate is at least 5% in the urine as hydroxytyrosol and tyrosol. Studies performed with animals indicate that phenol-rich olive oil reduces the oxidizability of low density lipoproteins (LDL) ex vivo or decreases urine markers of oxidative processes in the body [51]. However, many nutritional studies in humans have failed to demonstrate the observed in vitro results due to the lack of knowledge of the molecular targets and/or pathways implicated, and due to reduced bioavailability.

## 5. Epidemiological and Clinical Studies

Several epidemiological reports have evidenced that the Mediterranean diet is characterized by the lower prevalence of coronary heart diseases, neurological disorders and some types of cancer [52]. These therapeutic effects are results of high consumption of olive oil, culinary herbs and red wine. A systematic review and a meta-analysis including 23,340 controls and 13,800 patients concluded that olive oil intake exerts a protective role on cancer risk. However, the authors indicate that it remains unclear which components of olive oil are responsible for the beneficial effects [53]. Although epidemiological studies suggest beneficial effects derived from intake of characteristic foods of the Mediterranean diet on the occurrence of cancer, clinical trials focused on oleuropein do not yet exist. A first randomized trial (The Lyon Diet Heart Study) with 605 patients following a Mediterranean diet evidenced a decreased cancer risk after four-year follow up [54]. The PREDIMED was randomized, single-blind, controlled field trial (ISRCTN35739639) comprising 4282 women aged 60 to 80 years who were randomly allocated to a Mediterranean diet containing extra-virgin olive oil, with mixed nuts, or a control diet focused on reduce dietary fat [55]. After a 4.8 years follow-up, the intervention group treated with extra-virgin olive oil reported to have significant preventive effects against breast cancer.

## 6. Preclinical In Vivo Studies

Highly compelling evidence from experimental model indicated that oral administration of oleuropein induced completely regressed tumors in 9–12 days in mice [56]. There was a significant development of skin cancer at week 17 in mice upon chronic exposure to ultraviolet B irradiation. Tumor volume of mice treated both with extract of olive leaves (300 and 1000 mg/kg body weight) and oleuropein (25 mg/kg body weight) reduced notably in weeks 25 to 30 [57].

**Figure 2:**
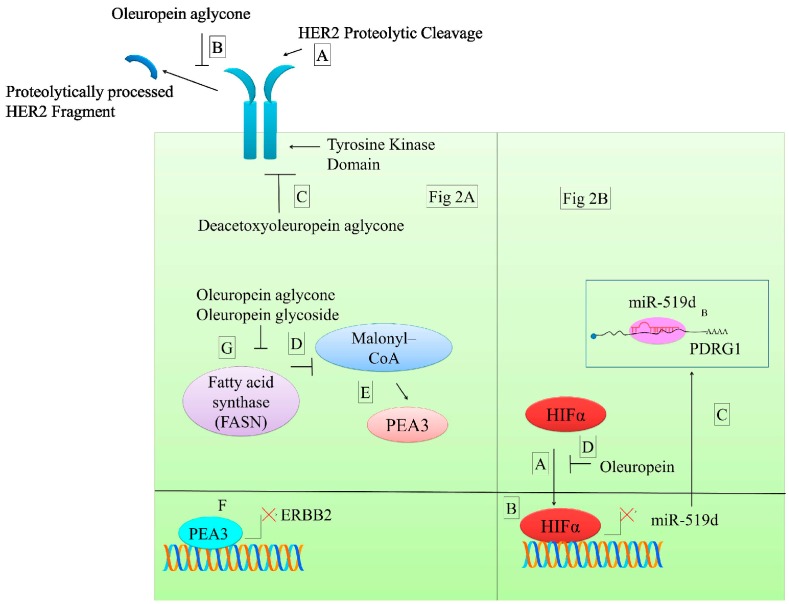
Various anticancer molecular mechanisms of oleuropein. (**A**) HER2 is proteolytically processed that is inhibited by oleuropein. Deacetoxyoleuropein aglycone inhibits activation of HER2. Malonyl-coenzyme A (CoA) plays a role in transcriptional repression of ERBB2 by facilitating entry of PEA3 in the nucleus. PEA3 binding sites are present within the promoter region of ERBB2. ERBB2 overexpressing breast cancer cells had lower levels of PEA3. As a result of oleuropein aglycone and oleuropein glycoside-mediated inhibition of FASN, higher levels of malonyl–CoA continue to be generated. Cytosolic accumulation of higher levels of malonyl-CoA triggered an increase and entry of PEA3 in the nucleus, where it occupied the PEA3 binding site and transcriptionally repressed ERBB2; (**B**) Hypoxia-inducible factor (HIFα) enters into the nucleus and transcriptionally represses miR-519d. miR-519d is involved in negative regulation of PDRG1 in cancer cells. However, treatment of cancer cells with oleuropein inhibits HIFα-mediated transcriptional repression of miR-519d and consequently miR-519d quantitatively inhibits PDRG1.

p53 and DNA damage-regulated protein 1 (PDRG1), an oncogene frequently overexpressed in different cancers, can be regulated by miRNA [58]. MiR-519d has been reported to post-transcriptionally modulate 3′-UTR of PDRG1 mRNA in nasopharyngeal carcinoma cells. Mutated 3′-UTR of PDRG1 transfected into miR-519d expressing cells revealed that mutant PDRG1 was not inhibited by miR-519d as evidenced by higher luciferase activity of mutated PDRG1 3′-UTR reporter. Hypoxia inducing factor (HIF1α) is involved in transcriptional downregulation of miR-519d by binding to hypoxia response elements (HRE) present in promoter region of miR-519d (Figure 2) [58]. Oleuropein inhibits the binding of HIF1α to miR19d promoter and consequently the expression if this microRNA is enhanced. It has been described that microRNA-519d inhibits the expression of PDRG1 [58].

## 7. In Vitro Anticancer Effects of Oleuropein

### 7.1. Effects on Human Epidermal Growth Factor Receptor (HER2 and HER1) Signaling

An abundance of experimental evidence emphasized on the potential involvement of HER2-induced signaling in cancer development and progression. HER2 is suggested to exist in functionally active form that interacts with the ligand-activated HER receptors. HER2 receptor is frequently overexpressed in breast cancer and drives activation of the phosphatidylinositol 3-kinase (PI3K)/Akt and/or mitogen-activated protein kinase (MAPK) pathways. In addition, HER2 interacts with other HER, such as HER1 (Erbb2, EGFR). HER2 is an effective therapeutic target for design and development of rationally designed drugs. Anti-HER2 monoclonal antibodies, such as trastuzumab, had attracted much attention of clinicians since HER2 is overexpressed in breast cancer patients. However, circumstantial evidence revealed that HER2-overexpressing breast cancer (BCa) cells developed resistance against trastuzumab. Different approaches are currently being tested to efficiently inhibit HER2 activation. Oleuropein aglycone (OA) is a remarkable candidate in this context [59]. It has been described that OA synergizes with trastusumab-sensitive breast cancer cell lines. The mechanism seems to be related to its role in the inhibition of the proteolytic processing of HER2 [59]. Trastuzumab (10 μg/mL) together with oleuropein aglycone (50 μM) downregulated HER2 up to 84% in serum-starved SKBR3 cells [59]. OA impressively improved trastuzumab efficacy >1000 times in BCa cells which had trastuzumab resistant phenotype. There is evidence which highlights upregulated expression of HER2 after treatment with high concentration of Trastuzumab. “HER2 super-expression” in BCa cells treated with trastuzumab was markedly suppressed oleuropein aglycone (100 μg/mL) [59] (Table 3).

Deacetoxyoleuropein aglycone (DAOA) acts as a tyrosine kinase inhibitor. Time-dependent decrease in HER2 tyrosine kinase activity was observed in DAOA-treated cancerous cells (Figure 2) [60]. In addition, cancer cells frequently present an increase of de novo synthesis of fatty acids. In order to sustain bioenergetics and structural demands in rapidly proliferating cells. OA and aglycone have been demonstrated to inhibit FASN and to increase the accumulation of malonyl-CoA that indirectly has been shown to inhibit transcription of Erbb2 (HER1) (Figure 2) [60].

### 7.2. Effects on MAPK Signaling

The MAPKs belong to a serine/threonine family of kinases which transduce the signals by post-translational modifications of different downstream effectors and transcription factors [65]. There has been a paradigm shift in the understanding of the MAPK pathway and it is now well recognized that these enzymes are able to respond to an array of stimuli to produce characteristically unique cellular outcomes [66]. These responses depend on the kinetics of their inactivation and activation, nuclear or cytosolic localization of the kinases, the complexes in which they are assembled, and the availability of substrates.

Several evidences propose that extracellular signal-regulated kinase (ERK), N-terminal kinase c-Jun (JNK), and p38 are the major trajectories of the MAPK signaling axis. For receptors with intrinsic tyrosine kinase activities (RTKs) and G protein–coupled receptors (GPCRs), the activation of MAPK cascade is triggered either through small GTP-binding proteins or adaptor proteins which transmit signal/s to MAP3Ks [66]. Keeping in view the fact that MAPKs are hierarchially assembled, MAP3Ks transfer the signals to downstream effector kinases via MAP2Ks which consequently activate of MAPK [66].

**Figure 3:**
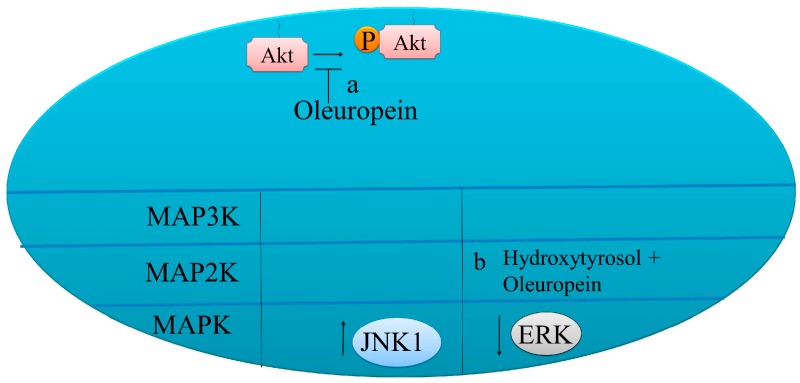
Pro-apoptotic molecular mechanisms of oleuropein in cancer cells. (**a**) Oleuropein mediated reduction of p-AKT levels. (**b**) Oleuropein enhanced p-JNK levels and reduced ERK1/2.

An increased level of phosphorylated JNK has evidenced to positively regulate apoptosis in HeLa cells. Oleuropein treatment increased apoptosis in HeLa cells through a mitochondrial apoptotic cascade derived from JNK activation [61] (Figure 3, Table 3).

G-protein-coupled estrogen receptor 1 (GPER) transduces the signals intracellularly by activating ERK1/2 in active cells. Ligand-binding and docking simulation techniques showed that oleuropein and hydroxytyrosol acted as agonists for GPER. Both compounds reduced SKBR3 cell growth and induced apoptotic processes via GPER-mediated activation of ERK1/2. Activation of ERK1/2 pathway was not observed in oleuropein-treated GPER silenced cells [63]. In addition, oleuropein and hydroxytyrosol also impaired 17b-estradiol-induced activation of ERK1/2 in breast MCF-7 cell line [62].

### 7.3. Oleuropein as an Apoptosis Inducer

The development of resistance against a wide ranging therapeutics and the loss of apoptosis underlie cancer progression. Apoptotic cell death is an extensively investigated aspect in molecular oncology. It is now well established that extrinsic (death receptor-mediated) or intrinsic pathway (mitochondrial) modulate apoptosis. Extrinsic signals, including tumor necrosis factor (TNF)-related apoptosis-inducing ligand (TRAIL), tumor necrosis factor-α (TNFα), and FasL, transduce the signals intracellularly through death receptors (DR4/DR5, TNFR, and Fas) and activate initiator caspases, such as caspase-8. These Cys-dependent aspartyl-specific proteases are centrally modulate apoptosis. Irradiation or chemotherapeutic agents initialized the mitochondrial pathway by activating BH3-only motif proteins such as BID. BID is proteolytically processed by caspase-8 and enters into mitochondria that facilitated the release of cytochrome *c*. B-cell lymphoma 2 (BCL-2)-associated X protein (BAX) and BCL-2 antagonist/killer (BAK) are pro-apoptotic proteins positioned at the outer mitochondrial membrane. tBID interacted with BAX and BAK and triggered their oligomerization [67].

Oleuropein is able to increase the levels of both BAX and cytochrome *c* in HeLa cells. Phosphorylated JNK was found to be necessary to induce apoptosis in HeLa cells. Oleuropein treatment induced an upregulation of p-JNK in HeLa cells. Accordingly, inhibition of JNK abrogated oleuropein-mediated apoptotic cell death in HeLa cells [61]. Expression levels of BAX and p53 (positive regulator of apoptosis) were increased in oleuropein-treated MCF-7 cells. Furthermore, Bcl2 (anti-apoptotic protein) was found to be reduced in oleuropein-treated cancer cells [68]. Oleuropein increased BAX and simultaneously suppressed Bcl2 in oleuropein-treated ER-negative breast cancer cells (SKBR3) [63] (Table 3).

Much more data will be forthcoming in the coming years with regard to oleuropein-mediated effects on expression of death receptors in different cancers. Moreover, combinatorial strategies using different other natural products or chemotherapeutic drugs may prove to be effective in restoring apoptosis in drug resistant cancer cell lines.

### 7.4. Effects on PI3K/AKT Signaling Axis

PI3K/AKT efficiently transmits the messages in the form of signals to downstream effectors. Accumulation of PIP_3_ facilitates the location of pleckstrin homology (PH) domain-containing proteins. Phosphoinositide-dependent kinase 1 (PDK1) and AKT protein kinase B (PKB) are proteins containing the PH domain.

Serine/threonine kinase mechanistic target of rapamycin (mTOR) exists as 2 multi-components nano-machineries, mTORC1 and mTORC2. mTORC2 promotes the AKT activity and stability in cancer cells. AKT is post-translationally modified by mTORC2 and PDK-1. Inhibition of AKT was noted to be an important step to induce apoptosis. Therefore, different research groups have used various natural products to inhibit AKT in cancer cells. There was a dose-dependent induction of apoptosis in oleuropein treated HepG2 human hepatoma cells. Oleuropein is reportedly involved in induction of pro-survival signals in cancerous cells that overexpressed AKT/PKB. AKT/PKB inhibition was essential to maximize oleuropein-mediated apoptosis (Figure 3) [69].

Combining oleuropein with AKT inhibitors will prove to be more useful in increasing apoptotic rate in tested cancer cell lines. Basal levels of phosphorylated ERK and phosphorylated AKT were found to be downregulated in thyroid cancer cells upon treatment with oleuropein [56]. Levels of phosphorylated AKT at 308th and 473th serine residues were considerably suppressed in prostate cancer cells (Figure 3) [70].

### 7.5. Regulation of Reactive Oxygen Species (ROS) Production

Oxidative stress is a comprehensively explored mechanism and has a vital role in regulation of different cellular functions. ROS can interact with and modify biological macromolecules such as lipids, proteins and DNA.

There was a dose-dependent reduction in endogenously generated ROS level in BCPAP and TPC-1 (thyroid cancer cells) upon treatment with oleuropein [56] (Table 3). On the contrary, ROS-modulatory effects exerted by oleuropein were different in prostate cancer cells (DU145). ROS production was markedly enhanced in DU145 cells after treatment with oleuropein. Oleuropein has also been isolated from *Zanthoxylum heitzii*. Co-treatment of HL-60 cells with bark extracts and fruits of *Z. heitzii* generated ROS that consequently induced apoptosis and cell death. Extracts of bark of *Z. heitzii* have been reported to induce a greater ROS production as compared to fruit extracts in HL-60 cells [58]. Nuclear factor-κB (NF-κB) and its main oncogenic target cyclin D1 were downregulated in ER-negative breast cancer cells upon treatment with oleuropein [64] (Table 3).

Future studies must focus on detection of in vivo redox status in multiple cancerous cells at different stage/s and in the non-transformed cells present in tumor micro-environment. This information will be helpful in development of small molecules that can locally target specific biochemical nodes.

### 7.6. Epigenetic Effects

Epigenetics refers to alterations in gene expression and chromatin organization, mainly derived from DNA methylation and histone modification but without changes in DNA sequence. Although it has been observed that extra virgin olive oils rich in secoiridoid can affect the acetylation and methylation state of histones, no study specifically analyzes the epigenetic effects of oleuropein. In one study, the treatment with extra virgin olive oils rich in secoiridoids allowed histones to remain in hyperacetylated states and derived from these processes may affect the expression of genes, leading to inhibition of cell cycle and notable decrease in viability of breast cancer cells [71]. Another investigation reported the stimulatory effect of extra virgin olive oil on type 1 cannabinoid receptor expression which was inversely correlated to DNA methylation at cannabinoid receptor type 1 gene promoter in Caco-2 cells but also in a rat model of colon cancer [72].

## 8. Conclusions

Oleuropein is a phenolic secoiridoid glycoside and one of the most abundant bioactive components contained in *Olea europaea*, which is known to modulate several oncogenic signalling pathways. The present review presents information from published literature about its anticarcinogenic and onco-suppressive effects, with an emphasis on molecular mechanisms implicated in cancer chemoprevention as well as therapeutic effects. Current research has shown that oleuropein acts as an anticancer agent by several major mechanisms, including targeting HER2, epigenetic modifications, interfering with MAPK pathway, modulation of apoptosis and PI3K/AKT signalling axis as well as by reducing ROS production in different cell types. Moreover, highly compelling evidence from preclinical studies has shown that oleuropein effectively induced complete regressed tumors mice experimental model. Regulation of microRNAs by oleuropein is also an insufficiently studied area that needs detailed research. How oleuropein modulates oncogenic and tumor suppressor miRNAs will be helpful in identifying potential targets of oleuropein. Deregulated intracellular signalling cascades have contributory role in cancer development and progression. However, breakthroughs related to oleuropein-mediated targeting of dysregulated signal transduction cascades have yet to be witnessed.

## Figures and Tables

**Table 1:** Various physical properties of oleuropein (CAS 32619-42-4) [19].

Physical Properties	Value
Molecular weight	540.5148 g/mol
Melting Point	88 °C
log P (octanol-water)	−0.080
Atmospheric OH Rate Constant	2.59 × 10^−10^ cm^3^/molecule-sec at 25 °C

**Table 2:** Concentration of oleuropein in different parts of the olive plant or in different products.

Part of the Plant/Product	Variety	Concentration	Units	References
Leaves	n.a.	22,640	mg/kg	[39]
	n.a.	0.21–5.06	g/kg·DW *	[33]
	n.a.	n.d.-173	mg/100 g	[40]
	Chemlali	4.32	g/100 g·DW	[41]
	Leccino	1.05	mg/g leaves	[42]
	Frantoio	3.19	mg/g leaves	
	Moraiolo	14.35	mg/g leaves	
	N2	7.06	mg/g leaves	
	N3 (Don Carlo)	8.39	mg/g leaves	
	Coratina	6.1	mg/g leaves	
	Kalamata	2.86	mg/g leaves	
	Nociara	3.7	mg/g leaves	
	I-77	3.03	mg/g leaves	
	Dritta	0.95–3.21	g/kg FW	[37]
	Leccino	0.80–2.44	g/kg FW	
	Caroleo	0.78–2.36	g/kg FW	
	Coratina	1.42–4.43	g/kg FW	
	Castiglionese	2.81–7.29	g/kg FW	
	Nebbio	3.27–8.61	g/kg FW	
	Grossa di Cassano	3.08–8.10	g/kg FW	
	n.a.	1685	mg/100g extract	[32]
Stem	Picual	523–651 (cortex stem)	mg/100g·FW	[43]
	Picual	320	mg/100 g·FW	[44]
Fruits	Arbequina	9.74–392 ^a,^** 3.63–475 ^b,^**	µmol/g dry pulp	[21]
	Arbequina	12–246 ^a,^** 18–320 ^b,^**	µmol/g dry pulp	
	Leccino	4.25	mg/g fruit	[42]
	Frantoio	3.48	mg/g fruit	
	Moraiolo	2.32	mg/g fruit	
	N_2_	2.26	mg/g fruit	
	N3 (Don Carlo)	5.78	mg/g fruit	
	Coratina	1.98	mg/g fruit	
	Kalamata	5.26	mg/g fruit	
	Nociara	9.96	mg/g fruit	
	I-77	6.83	mg/g fruit	
Fruits—flesh (pepper stuffed)	Manzanilla	147	mg/kg	[45]
Fruits—flesh (anchovy stuffed)	Manzanilla	104	mg/kg	
Fruits—whole by-product (pepper stuffed)	Manzanilla	148	mg/kg	
Fruits—whole by-product (pepper stuffed)	Manzanilla	199	mg/kg	
Stone (without seed)	Manzanilla	750	mg/kg	
Seed	Manzanilla	569	mg/kg	
Roots	Picual	140	mg/100g·FW	[44]
Virgin olive oil	La Pepa	140	mg/kg	[46]
	Severini	120	mg/kg	
Olive oil pomace	La Pepa	83	mg/kg	
	Severini	82	mg/kg	
Diettary supplements (Bonoolive^®^)		100	mg/one dosage unit	[34]

^a^ Organic farming; ^b^ conventional farming; * depends on irradiation and hot water blanched treatments; ** depends on the time of the year; DW, Dry weight; FW, Fresh weight; n.a., data not available, n.d., not detected.

**Table 3:** Anticancer effects of Oleuropein and its derivatives.

Bioactive Ingredient	Mechanisms	Cancer Cells	Reference
Oleuropein aglycone	HER2 proteolytic processing	Breast cancer cells	[59]
Deacetoxyoleuropein aglycone	HER2 kinase inhibition	Breast cancer cells	[59]
Oleuropein glycoside Oleuropein aglycone	Decrease in fatty acid synthase (FASN); Increase in malonyl–CoA	Breast cancer cells	[60]
Oleuropein	Increase in phosphorylated JNK level	HeLa	[61]
Oleuropein hydroxytyrosol	Inhibited activation of ERK1/2	MCF-7	[62]
Oleuropein	Increase in Bax, cytochrome c	HeLa	[61]
Oleuropein hydroxytyrosol	Reduce Bcl-2Activate GPER/GPR30-dependent pathways	SKBR3 breast cancer cells	[63]
Oleuropein	Reduce phosphorylated AKT	Thyroid cancer cells	[56]
Oleuropein	Suppress NF-κB and cyclin D1	ER-negative breast cancer cells	[64]
